# Juvenile Idiopathic Arthritis

**DOI:** 10.4274/balkanmedj.2017.0111

**Published:** 2017-03-28

**Authors:** Kenan Barut, Amra Adrovic, Sezgin Şahin, Özgür Kasapçopur

**Affiliations:** 1 Department of Pediatric Rheumatology, İstanbul University Cerrahpaşa Medical School, İstanbul, Turkey

**Keywords:** juvenile idiopathic arthritis, biologic therapy in childhood, pediatric rheumatology, chronic arthritis in childhood

## Abstract

Juvenile idiopathic arthritis is the most common chronic rheumatic disease of unknown aetiology in childhood and predominantly presents with peripheral arthritis. The disease is divided into several subgroups, according to demographic characteristics, clinical features, treatment modalities and disease prognosis. Systemic juvenile idiopathic arthritis, which is one of the most frequent disease subtypes, is characterized by recurrent fever and rash. Oligoarticular juvenile idiopathic arthritis, common among young female patients, is usually accompanied by anti-nuclear antibodie positivity and anterior uveitis. Seropositive polyarticular juvenile idiopathic arthritis, an analogue of adult rheumatoid arthritis, is seen in less than 10% of paediatric patients. Seronegative polyarticular juvenile idiopathic arthritis, an entity more specific for childhood, appears with widespread large- and small-joint involvement. Enthesitis-related arthritis is a separate disease subtype, characterized by enthesitis and asymmetric lower-extremity arthritis. This disease subtype represents the childhood form of adult spondyloarthropathies, with human leukocyte antigen-B27 positivity and uveitis but commonly without axial skeleton involvement. Juvenile psoriatic arthritis is characterized by a psoriatic rash, accompanied by arthritis, nail pitting and dactylitis. Disease complications can vary from growth retardation and osteoporosis secondary to treatment and disease activity, to life-threatening macrophage activation syndrome with multi-organ insufficiency. With the advent of new therapeutics over the past 15 years, there has been a marked improvement in juvenile idiopathic arthritis treatment and long-term outcome, without any sequelae. The treatment of juvenile idiopathic arthritis patients involves teamwork, including an experienced paediatric rheumatologist, an ophthalmologist, an orthopaedist, a paediatric psychiatrist and a physiotherapist. The primary goals of treatment are to eliminate active disease, to normalize joint function, to preserve normal growth and to prevent long-term joint damage. Timely and aggressive treatment is important to provide early disease control. The first-line treatment includes disease-modifying anti-rheumatic drugs (methotrexate, sulphasalazine, leflunomide) in combination with corticosteroids, used in different dosages and routes (oral, intravenous, intra-articular). Intra-articular application of steroids seems to be an effective treatment modality, especially in monoarthritis. Biological agents should be added in the treatment of unresponsive patients. Anti-tumour necrosis factor agents (etanercept, infliximab, adalimumab), anti-interleukin-1 agents (anakinra, canakinumab), anti- interleukin-6 agents (tocilizumab) and T-cell regulatory agents (abatacept) have been shown to be safe and effective in childhood patients. Recent studies reported sustained reduction in joint damage with even complete clinical improvement in paediatric patients, compared to previous data.

## JUVENILE IDIOPATHIC ARTHRITIS

Juvenile idiopathic arthritis (JIA) is the most common chronic rheumatic disease in childhood and is of unknown aetiology. JIA encompasses several different subgroups and predominantly presents with peripheral arthritis. The disease spectrum consists of various clinical conditions. Endogenous and exogenous antigens with increased inflammatory response have been shown to play a central role in the pathogenesis of the disease. Chronic inflammation limits the daily activities and productivity of the patient. By definition, disease onset prior to the age of 16 years and arthritis persisting for longer than 6 weeks are required criteria for diagnosis of JIA (1,2). JIA represents a new, common name for several conditions considered separately in the past. Furthermore, similar terms were previously used for the same clinical entity: juvenile rheumatoid arthritis and juvenile chronic arthritis (JCA) (3). The aim of this manuscript is to review the main disease characteristics of, and to update novel therapeutical options for, the most common chronic rheumatic disease in childhood.

### Classification

The International League of Associations for Rheumatology (ILAR) proposed classification criteria for JIA. The last revised criteria, which were updated in 2001, are still widely used. According to ILAR classification criteria, JIA is divided into seven subtypes: oligoarticular JIA, seropositive polyarticular JIA, seronegative polyarticular JIA, systemic-onset JIA (sJIA), enthesitis-related arthritis (ERA), juvenile psoriatic arthritis (JPsA) and undifferentiated JIA (Table 1) (4). The main criteria of the disease include disease onset prior to the age of 16 and arthritis of at least one joint persisting for longer than 6 weeks with exclusion of any other possible cause of joint inflammation. The disease subtype should be assessed at the onset of the disease and during the follow-up. Initial classification is made according to the clinical features of the first six months in the course of the disease. The onset of new clinical features during the course of the disease determines the final disease subtype (1). The main objective of the disease subclassification is to homogenize the disease groups, define the therapy options, choose the follow-up strategies and predict the disease prognosis.

### Epidemiology

The epidemiological characteristics of the disease are important in order to establish the influence of genetic and environmental factors on the course of the disease. Additionally, they could give a clue as to the correct treatment approach and improve the preventive health-care methods. The prevalence of JIA varies from region to region. Despite the miscellaneous studies on JIA, the disease prevalence and incidence remain unclear, due to the lack of uniform classification methods and the diversity of disease frequencies in different regions. Data from literature suggest a disease incidence of 1-22 in 100.000 and a disease prevalence of 7-150 in 100.000 (5,6,7). A study from Turkey reported a prevalence of chronic arthritis in childhood of 64 in 100.000 (8). Interestingly, a study from Australia showed a prevalence as high as 400 in 100.000 (9).

### Aetiopathogenesis

The aetiopathogenesis of the disease is still unclear. The most acceptable theory supports the influence of immunogenic mechanisms secondary to different genetic and environmental factors. Infections, together with stress and trauma, are considered to be the most responsible aetiological factors (6). Gut microbiota is emerging as a relevant factor of autoimmune diseases, including JIA, according to recent studies (10). The increased frequency of autoimmune diseases among JIA patients suggests the genetic basis of the disease (11). Human leukocyte antigen (HLA) B27 and the other HLA tissue types are the most commonly mentioned genetic factors (3,12,13,14,15). Various infections are considered to be responsible for JIA pathogenesis: enteric infections, parvovirus B19, rubella, mumps, hepatitis B, Epstein-Barr virus, mycoplasma and chlamydia infections (2,16). Potential trigger-induced T-lymphocytes and secreted cytokines lead to joint destruction. Macrophages, induced by secreted mediators, produce pro-inflammatory cytokines [interleukin (IL) 1, IL-6, tumour necrosis factor (TNF)-α]. Thus, the acute phase markers [C-reactive protein (CRP), erythrocyte sedimentation rate (ESR)] increase and the acute inflammation of joints occurs with an increase in synovial fluid. Synovial inflammation (synovitis) is characterized by villous hypertrophy and hyperaemia of the subsynovial tissue. Synovial hypertrophy and synovitis secondary to chronic inflammation are known as “panni”. The T-lymphocyte percentage in synovial fluids varies among different JIA subtypes, possibly explaining the difference in treatment response among JIA subgroups (6,7,17).

## JUVENILE IDIOPATHIC ARTHRITIS SUBTYPES

### Systemic JIA

**Figure 1:**
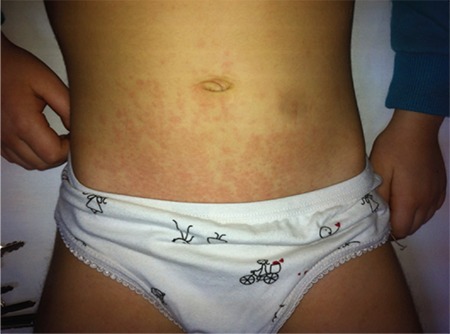
Typical rash in systemic juvenile idiopathic arthritis.

This disease subtype, characterized predominantly by systemic symptoms, affects both female and males with the same frequency and may occur at any time during childhood. The presence of arthritis and intermittent fever for at least 2 weeks plus one of the following defines the disease: typical rash, generalized lymphadenopathy, hepatosplenomegaly or serositis. Although its frequency differs between different geographical regions, systemic JIA accounts for 10-20% of total JIA (6). The temperature rises up to 39.5 °C once or twice in a day. The intermittent fever is generally accompanied by a typical, salmon pink-coloured rash, which commonly occurs on the trunk and proximal extremities and vanishes with the decline of the fever (Figure 1). Occasionally, polyarticular arthritis, including both large and small joints, may appear during the course of the disease (18). In general, systemic symptoms of fever and rash resolve after the emergence of polyarthritis, which makes distinguishing it from regular polyarticular JIA challenging (3). Fever and other systemic symptoms can persist for months but rarely longer than 6 months. Hepatosplenomegaly and lymphadenopathy are seen in about one-third of patients. Serositis, including pericarditis and pleuritis, may be seen, presenting with marked chest pain. Abdominal pain and myalgia may appear during the peak of the fever (19,20). Leukocytosis, hypochromic microcytic anaemia, thrombocytosis, acute-phase reactant elevation and an increased level of transaminases may be noticed. Elevation of the ferritin level is a relevant issue for sJIA. Auto-antibodies, ANAs and rheumatoid factor (RF) are negative (19). Possible complications of sJIA include osteopenia, osteoporosis, growth retardation, erosive arthritis and amyloidosis. Macrophage activation syndrome (MAS), a severe, life-threatening sJIA complication, is seen in 5-8% of cases. It is associated with serious morbidity and mortality. Moderate/severe disseminated intravascular coagulation features (thrombocytopenia, increased fibrin degradation products, markedly elevated levels of D-dimers, prolonged prothrombin time and partial thromboplastin time) are present. The ESR drops sharply in association with hypofibrinogenaemia. Typically, liver enzymes, lactate dehydrogenase (LDH), triglycerides and ferritin levels are elevated, sometimes with extreme hyperferritinaemia, hypoalbuminaemia and hyponatraemia (21). A European League Against Rheumatism/American College of Rheumatology/Paediatric Rheumatology International Trials Organisation collaborative initiative recently proposed criteria for MAS complicating systemic JIA: febrile patients with confirmed or suspected sJIA with ferritin >684 ng/mL and any two of the following - platelet count ≤181x109/L, aspartate aminotransferase >48 units/L, triglycerides >156 mg/dL, fibrinogen ≤360 mg/dL. Although demonstration of prominent haemophagocytosis in the bone marrow aspiration represents a valuable finding, it has not been included in MAS diagnostic criteria since haemophagocytosis cannot be documented in the early stage of MAS (22). The diagnosis of sJIA may be difficult, especially in the early phase of the disease without apparent arthritis. Many conditions should be considered in differential diagnosis of sJIA: infections (septicaemia, bacterial endocarditis, brucellosis, typhoid fever, leishmaniasis, viral infections), malignancies (leukaemia, lymphoma, neuroblastoma), rheumatic fever, connective tissue diseases, Kawasaki disease, Castleman’s disease and autoinflammatory diseases (20). Differentiating sJIA at disease onset from Kawasaki disease can be challenging because they have many common clinical and laboratory features, albeit prolonged fever and arthritis are more specific for sJIA (23).

### Oligoarticular Juvenile Idiopathic Arthritis

Oligoarticular JIA is the most common JIA subtype in developed countries, and is generally seen among female patients younger than six (1). It is further subdivided into two subgroups: persistent (no more than four joints affected during the course of the disease) and extended (after the initial 6-month period, the total number of affected joints exceeds four) (4). The RF is negative but the ANA is positive in 70-80% of patients. Uveitis risk is found to be higher in ANA-positive patients (5,24). Uveitis, rather than arthritis, is the main disability factor in that patient group. Oligoarticular JIA predominantly involves lower-extremity joints, such as the knee and ankle joint. The hip joint is rarely affected. Small-joint involvement is pretty rare in this entity (6). Since small-joint involvement could be an early sign of psoriasis, the family history in particular should be assessed in this group (25). Typically, the disease presents with monoarthritis with an excellent prognosis and without any functional disability. The onset of symptoms could be sudden or insidious. Morning stiffness could present at the onset of the disease and if untreated becomes more prominent in the further course of the disease and even limping could be seen. The affected joint is swollen and often warm, but usually not very painful or tender. These children do not have systemic signs in general (3,26). Laboratory indicators of inflammation may be normal, although mild to moderate elevation of the ESR and CRP levels may occur during the acute phase of the disease. Elevated ESR and arthritis of upper extremities are more common in patients with extended oligoarticular JIA. Mild anaemia and leukocytosis could be seen in patients with acute arthritis (26,27). The disease is characterized by a benign clinical course, in general. However, erosions due to extension to polyarthritis and uveitis are two of the possible severe complications. A significant difference in length between extremities could be seen, depending on the extent of the joint damage. Growth retardation is rare. Although remission is achieved frequently, disease flares may occur many years later; thus, regular follow-up for at least 4-5 years is mandatory (26).

### Polyarticular Juvenile Idiopathic Arthritis

**Figure 2:**
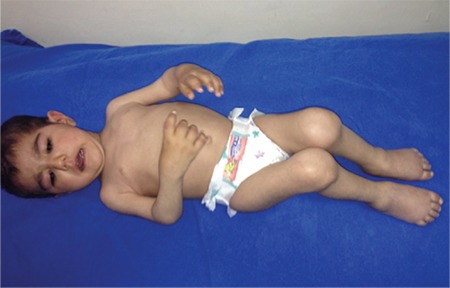
Widespread joint involvement in polyarticular juvenile idopathic arthritis.

Polyarticular JIA is defined as arthritis of five joints or more during the first 6 months of the disease. The disease is divided into two subgroups, according to RF positivity. The frequency of the disease varies in different geographical regions with the approximate frequency of RF-negative polyarticular JIA being 11-30 % and that of RF-positive being 2-10% (6,20,28). Both of the disease subgroups are more common among girls. RF-negative polyarticular JIA displays a biphasic trend with peaks of onset between 2 and 4 years and 6 and 12 years. The RF-positive subgroup is more common in later childhood and adolescence (20). Mild fever, weight loss and anaemia are seen in both disease subgroups. Furthermore, moderate hepatosplenomegaly and mild growth retardation could develop (3). Seronegative polyarticular JIA is an entity consisting of three different subgroups. The first one includes an oligoarticular JIA-like condition with the involvement of more than four joints in the first 6 months; the main characteristics of the disease are increased uveitis risk, the development of asymmetric arthritis, early disease onset, female predominance, ANA positivity and association with HLA-DRB1*0801 (20). The second disease subgroup mimics the RF-negative polyarticular JIA with early disease onset, symmetrical involvement of both small and large joints, ANA negativity and variation in disease prognosis. The third disease subtype shows the worst prognosis with poor response to treatment and frequent disease sequelae (20). From time to time, the disease can appear with oligoarticular involvement extending to polyarthritis. The arthritis of the small joints of upper extremities, metacarpal-phalangeal joints and the wrist is typically symmetric (Figure 2). The arthritis of the small joints of lower extremities could be seen, albeit less frequently. Involvement of the hip, cervical spine and shoulder could be seen as well. Temporomandibular arthritis is present in the majority of patients, resulting in secondary microretrognathia (3,6,29). These disease subtypes are associated with a high rate of damage, especially hip joint involvement, which is related to high morbidity and surgical interventions (30). Although large-joint involvement could be seen, symmetrical small-joint arthritis is more typical. The clinical course of the RF-positive polyarticular JIA reminds us of adult RA (20). Subcutaneous nodules, histologically similar to nodules seen in RA, could also be seen in RF-positive patients. These nodules spontaneously resolve and are parallel with serum RF levels. Positivity of RF and anti-CCP are the most relevant predictors of significant joint damage (31). Middle anaemia could be documented, due to chronic inflammation in polyarticular JIA patients. Lymphadenopathy and hepatosplenomegaly could accompany the other disease features during the active-disease period. Growth and development retardation may complicate the disease, depending on disease activity. The transaminase level could increase due to disease activity or secondarily to hepatotoxicity of the therapy. The level of transaminases decreases parallel with disease remission (3).

### Enthesitis Related Arthritis

ERA has been one of the most controversial topics in paediatric rheumatology for the last 25 years. These patients show characteristics of both JIA and juvenile spondyloarthropathies. For years, different names were used for the same clinical entity, including type 2 oligoarticular JIA, JCA with late onset, seronegative enthesopathy and arthropathy, arthropathy associated with HLA B27 or juvenile spondyloarthropathy with early onset. The term ERA is defined by Durban classification (3,4,13). The disease is typically seen among males, after the age of six. The main characteristics of the patients include RF and ANA negativity with findings of enthesopathy and asymmetric arthritis of the lower extremities. HLA B27 positivity is reported in 65-80 % of patients (13). Enthesopathy represents the inflammation of the attachment sites of the tendons to the bones. Weiss et al. (32) reported enthesopathy in a single site in 47% and in three different sites in 18% of patients during the same clinical examination. The Achilles tendon is the most commonly affected site. Patellar insertion of the quadriceps tendon, and calcaneal and metatarsal insertions of the plantar fascia could be affected as well. The affected site is characterized by pain and sensitivity. However, enthesopathy can be seen in different conditions, such as familial Mediterranean fever, Behçet’s disease, Osgood-Schlatter syndrome and fibromyalgia (12,13). Typical joint involvement is asymmetric oligoarticular lower-extremity arthritis with the knee and ankle being most commonly affected. It is thought that different infections or traumas could trigger the disease. The most significant distinction from oligoarticular JIA is the involvement of the hip joints. Prolonged arthralgia of the lower extremities could be seen at disease onset. This phase of the disease is not characterized by axial skeleton involvement. The response to non-steroidal anti-inflammatory drugs is excellent with complete or partial remission. The risk of sequelae is low (12,13). However, data from literature show that ineffective treatment in childhood leads to disease progression and the development of ankylosing spondylitis seen among adults. Consequently, early diagnosis and timely treatment represent one of the most relevant topics in paediatric rheumatology (12).

### Juvenile Psoriatic Arthritis

JPsA is still an important topic of discussion in paediatric rheumatology. According to the ILAR criteria, the disease is defined by arthritis together with either a psoriatic rash or two of the following: dactylitis; nail pitting or onycholysis; psoriasis in a first-degree relative (20). Since the articular involvement generally occurs a few years before the development of skin manifestations, the diagnosis could be challenging. Articular involvement varies from symmetrical small-joint arthritis to asymmetrical lower-extremity large-joint arthritis and finally may progress to polyarthritis mimicking seropositive rheumatoid arthritis. Distal interphalangeal joint involvement typically suggests psoriatic arthritis. In general, arthritis of metacarpophalangeal, proximal interphalangeal and distal interphalangeal joints of one or more fingers forms the “sausage-like” fingers known as dactylitis (3,6,33). Features of ERA (enthesitis, sacroiliitis, spondylitis etc.) can be seen in some patients.

Typically, psoriatic plaques are seen on the extensor sides of joints, haired skin, the umbilicus and the perineum. Nail changes, including nail dystrophy, subungual hyperkeratosis and onycholysis are common among patients with psoriasis, albeit being less frequent in patients without arthritis (3,20,33). Increased acute phase markers, anaemia of chronic disease and thrombocytosis could be seen. ANA is found in low or moderate titres in a significant proportion of patients. HLA B27 positivity accounts for 30% (33).

### Juvenile Idiopathic Arthritis and Uveitis

Since uveitis represents one of the most severe extra-articular manifestations of JIA, regular screening by an experienced ophthalmologist is of crucial importance. The frequency of JIA-associated anterior non-granulomatous uveitis (iridocyclitis) is reported as being 15-67% in various European regions (34). More attention should be paid to ANA-positive oligoarticular JIA female patients with early disease onset. Since these patients are at an especially high risk of uveitis, the ophthalmological examination should be performed every three months (6). The most frequently reported type of uveitis is chronic anterior uveitis (68.3%), followed by acute anterior disease (16.2%), recurrent anterior disease (12%) and panuveitis (3.5%). Among 1081 JIA cases, the uveitis percentage was reported as 13.1% (35). The onset of uveitis in oligoarticular JIA patients is often insidious and usually asymptomatic, although half of the children could have some symptoms related to uveitis (pain, redness, headache, photophobia, change in vision) (6). Patients with polyarticular JIA show no signs of ocular involvement at disease onset but 5-10% of them develop uveitis during the course of the disease (6). Acute, symptomatic uveitis is seen in 10-20% of ERA patients. Uveitis is characterized by attacks of unilateral or bilateral acute, painful, photophobic iritis and with hyperaemia of the sclera and conjunctiva. Patients with HLA B27 positivity are more prone to developing acute anterior uveitis (6,34). Diagnosis and treatment delay lead to severe complications including band keratopathy, cataract, glaucoma or blindness. The frequency of blindness secondary to uveitis has markedly decreased due to new developments in diagnosis and treatment in recent studies (34,35). Although the frequency of ANA positivity among patients with uveitis has been reported as being as high as 90% in countries of Western Europe, studies from Turkey showed its percentage to be lower (36). The initial treatment approach consists of local or systemic glucocorticoids. In order to avoid the adverse effects of glucocorticoids or, in unresponsive patients, different agents should be added to the therapy: methotrexate, azathioprine, mycophenolate mofetil and cyclosporine A. Biological agents are used in patients with resistant disease (infliximab, adalimumab, tocilizumab). Surgical interventions are reserved for patients with disease complications (37).

### Diagnosis and Disease Activity Measurements

The diagnosis of JIA is based upon clinical criteria (Table 1). Occasionally, it can take time to establish the definitive diagnosis of the disease, due to the insidious course of the disease. Since there are no laboratory data specific for the disease, patients could be misdiagnosed at disease onset. Laboratory features are useful in differential diagnosis and in defining the disease subclassification (3).

The other relevant issue in the follow-up of JIA patients is the measurement of disease activity. Various measures are used in the assessment of disease activity (e.g. parent/patient visual analogue scale, physician visual analogue scale, number of active joints, anaemia, thrombocyte count, ESR, Steinbrocker score etc.) but none of them has been shown to be totally accurate. Many different instruments for measuring JIA disease activity have been established: Juvenile Arthritis Functional Assesment Report, Juvenile Arthritis Functional Assesment Scale, Juvenile Arthritis Disease Activity Score (JADAS). Furthermore, Childhood Health Assesment Questionnaire (CHAQ) and Paediatric Quality of Life Inventory were developed with the intention of incorporating estimates of physical, social and mental functioning into health assessment. Among numerous available instruments for JIA, CHAQ and JADAS are most widely used in routine practice. The American College of Rheumatology proposed measurements consisting of five items that are used in the assessment of disease activity in JIA patients (Table 2) (38,39,40,41,42,43,44).

### Treatment

Chronic inflammation of the joints markedly limits the patient’s functional ability and productivity in daily life. The underlying reason for the mentioned complications is the inflammation that is not under control. Other than joint-related complications, many other complications could develop in untreated patients, including growth retardation, uveitis, blindness and life-threatening MAS. Adverse effects of drugs should be kept in mind as well (e.g. osteoporosis, growth retardation secondary to glucocorticoids etc.) (6). Thus, the JIA treatment should be prompt and effective. The importance of supportive measurements, such as adequate nutrition, calcium and Vitamin D supplements, should not be underestimated (7,45,46). The aim of the therapy should be multidimensional: to control the pain, to preserve the range of motion/muscle strength/muscle function, to induce disease remission, to manage systemic complications and to facilitate normal physical and psychosocial development. The duration of treatment should be adjusted every 3 months, until the goal of treatment is achieved. The disease activity should be regularly assessed (every 1-6 months). Different instruments are used in disease activity assessment (Table 2). Disease activity indexes should be used in determining treatment goals. Additionally, compliance of the patient and parent, JIA subtype and treatment-related side effects should not be ignored in optimizing the best treatment target. Children and parents should be informed in detail about the process and goals (45).

Randomized controlled studies in paediatric rheumatology during the last 10-15 years have revealed the efficacy and safety of various agents. Table 3 shows the drugs commonly used in JIA treatment.

## NON-BIOLOGICAL MEDICAL TREATMENT

### Non-Steroidal Anti-Inflammatory Drugs

Non-steroidal anti-inflammatory drugs (NSAIDs) represent the traditional initial approach. Ibuprofen, indomethacin, tolmetin and naproxen are the most commonly used agents. This group of drugs is used particularly in children under 12 years old. In patients with oligoarticular JIA the disease remission could be induced by NSAIDs. The main characteristics of the drugs are their analgesic effect in lower doses and anti-inflammatory effect when used in higher doses. Treatment response is seen in the first 1-3 days with pain relief. This type of drug is considered tolerable in childhood patients, despite the abdominal pain and headache that could occasionally be seen as an adverse effect (45,47).

### Corticosteroids

This group of drugs is characterized by the most potent anti-inflammatory activity. However, the usage is limited due to numerous side effects and low efficacy in the prevention of joint destruction. Intra-articular administration (methylprednisolone acetate, triamcinolone hexacetonide) has been shown to be effective in inducing remission in oligoarticular JIA patients, even with a single injection (48). Oral or parenteral administration of steroids has the ability to abate systemic symptoms in patients with the systemic form of the disease. Symptoms such as joint pain, swelling, sensitivity or disease-related carditis, hepatitis, lung disease and fever show a significant response to steroid treatment. However, due to numerous side effects, usage in low doses or on alternative days is recommended in patients in whom control of the disease has been achieved. The generally used dose of steroids is up to 1 mg/kg/day. The dose could be increased up to 1-2 mg/kg/day in patients with cardiac insufficiency or tamponade secondary to carditis or pericarditis. Patients with severe clinical presentation of systemic JIA should be treated with a high dose of steroids (30 mg/kg/day) for three consecutive days (45,47).

## DISEASE MODIFYING ANTI-RHEUMATIC DRUGS

### Methotrexate

Methotrexate is a folate antagonist, proven to be quite an effective and safe drug with long-lasting activity. The recommended treatment dose is 10-15 mg/m2/week or 0.5-1 mg/kg/week. Most patients show a response in the first 2-3 weeks of treatment. Occasionally, it can take some time to achieve treatment response. Folic acid or folinic acid at a dose of 1 mg/kg/day is used in order to reduce the adverse effects including bone marrow suppression, nausea, oral ulcerations and hair loss. The fact that folinic acid decreases the methotrexate activity should not be forgotten (6,45).

### Sulphasalazine

Different studies have reported the efficiency of sulphasalazine in patients with JIA, especially in the oligoarticular- and enthesitis-related forms of the disease. Treatment response is achieved in 6-8 weeks of treatment, in general. Headache, rash, gastrointestinal toxicity, myelosuppression, hypogammaglobulinaemia and allergic reactions are some of the possible adverse effects. The initial dose is 10-20 mg/kg/day, gradually increasing to 50 mg/kg/day in the following few weeks (6,49).

### Other DMARDs Used in JIA

Leflunomide is a pyrimidine synthesis inhibitor used in cases of methotrexate intolerance. Previous studies showed leflunomide to be equivalent to methotrexate in JIA treatment (50). Cyclosporine-A is the calcineurin inhibitor commonly used in patients with systemic JIA-associated MAS. It has no effect on joint damage (47).

### Biological treatment

The advance in biologic therapeutics over the past 15 years has led to marked improvement in JIA treatment. In the biological era, the rate of joint damage decreased, and achieved disease remission increased with an increased number of patients with inactive disease. Despite the promising results of these medications, the blockade of important immunological pathways necessitates detailed safety monitoring. However, previous studies reported the safety of biological agents. The efficacy and safety of the biological agents used in JIA treatment are explained in the following text.

**Etanercept:** Etanercept is a fusion protein that binds to soluble TNFα, decreasing downstream TNF receptor-mediated signlaling. Etanercept is a biological agent with relevant efficacy in the treatment of peripheral arthritis. It has proved to be the most efficient treatment option in polyarticular JIA patients. The dose of the drug is 0.8 mg/kg/week. The efficacy of etanercept becomes more prominent after the second or third dose (45,51). The most common adverse effect is the local reaction at the site of injection. Thus, the drug should be administered to different parts of the body. Recurrent upper respiratory tract infections are less frequent while severe infections requiring hospitalization are extremely rare (52,53).

**Infliximab:** Infliximab is a chimeric monoclonal antibody that has a high affinity for TNFα. Unlike etanercept, infliximab binds to both soluble and membrane-bound TNFα. Its efficacy in JIA therapy has been shown previously. The dose in usage is 3-6 mg/kg/4-8 weeks (maximum dose 200 mg). Spondyloarthropathies, inflammatory bowel disease, psoriatic arthritis and uveitis show a particularly excellent response to infliximab treatment (37,45,54). Combined use of infliximab with methotrexate markedly increases drug efficiency. Apart from mild upper respiratory system infections, the frequency of severe and opportunistic infections is unremarkable. Compared to other TNF antagonists, allergic reactions during i.v. infusion appear to be more common (45,53).

**Adalimumab:** Adalimumab is a fully humanized monoclonal antibody that binds to TNFα. The usual dose is 24 mg/m^2^/15 days (maximum 40 mg). Combined usage of adalimumab with methotrexate enhances the drug activity. It has been shown to be an efficient and safe drug as a first or second line of JIA therapy (1,55).

**Anakinra:** Anakinra is a recombinant human IL-1 receptor antagonist. It is administered subcutaneously in doses of 2-10 mg/kg/day (maximum 200 mg). Since IL-1 plays an important role in the pathogenesis of sJIA, multicentric studies showed the efficacy and safety of anakinra in sJIA treatment (56). Occasionally, injection pain and local injection site reactions make its usage difficult. In general, it is a well-tolerated drug with severe infections seen rarely (45,52).

**Canakinumab:** Canakinumab is a fully humanized monoclonal antibody that binds specifically to IL-1β. A multicentric study reported its efficacy in patients with systemic JIA (57). The recommended dose is 4 mg/kg/every 4-8 weeks for children under 40 kg in weight and 150 mg every 4-8 weeks for children above 40 kg. Canakinumab is used in many auto-inflammatory conditions. Due to a longer half-life and less frequent local reactions at the injection site compared to anakinra, it is commonly chosen as a first-line therapy by clinicians (45). Previous studies reported a slightly higher frequency of infections in treated patients, compared to placebo (52).

**Tocilizumab:** Tocilizumab is a recombinant, humanized monoclonal antibody that binds to the IL-6 receptor. It has been commonly used in active sJIA patients older than two, alone or in combination with methotrexate. The recommended dosage is 12 mg/kg/2-4 weeks below 12 kg and 8 mg/kg/2-4 weeks above 12 kg. Tocilizumab is used in the treatment of unresponsive systemic JIA patients, particularly in those with active arthritis that show no improvement and in polyarticular JIA patients (45,58). Double-blind, placebo-controlled studies with tocilizumab showed no significant increase in infections and no cases of tuberculosis or other opportunistic infections. It is important to mention that some infections could remain unrecognized in patients under tocilizumab treatment, due to its ability to decrease acute-phase markers and to inhibit fever reactions (45,52).

**Abatacept:** Abatacept is a recombinant fusion protein that downregulates T-cell stimulation, leading to decreased B-cell and macrophage activation. The drug is used as monthly injections at a dose of 10 mg/kg. Its efficiency and safety in patients with polyarticular JIA has been reported in previous studies. Unresponsiveness to other anti-TNF agents is an indication for abatacept. Except for mild infections requiring no hospital treatment, severe opportunistic infections have not been documented in patients treated with this drug (45,52,59).

**Rituximab:** Rituximab is a human monoclonal antibody that increases B-cell apoptosis and decreases mature B-cells carrying CD20. Since B-cells represent its main target, it is shown to be eficient in all conditions related to B lymphocytes. The proposed dose of this drug is 375 mg/m^2^ for three or four doses. Rituximab has been used in paediatric lupus erythematosus patients as off-label. Data on the administration of rituximab in JIA patients are limited. There is a study reporting its efficacy in JIA (60). Vaccination for encapsulated bacteria is mandatory prior to treatment with rituximab (45,60).

**Tofacitinib/CP-690.550:** Tofacitinib/CP-690.550 is a selective JAK inhibitor that acts by inhibiting JAK 1, JAK 2 and STAT 1. Currently, tofacitinib is used in the treatment of adult RA. There is an ongoing open-labelled study on tofacitinib in JIA treatment (45).

### Prognosis

Novel biological therapeutics, early aggressive treatment and effective intra-articular administration of steroids have led to marked improvement in the prognosis of JIA. Despite this progress, some patients still have an active disease with a progressive clinical course. Although the majority of oligoarticular JIA patients achieve remission, some of them still do not resolve totally (5,26). The risk of uveitis, joint erosions with the development of severe sequelae and extension of the disease to polyarticular disease makes oligoarticular JIA an entity that requires careful monitoring with a need for aggressive, early treatment in cases of higher risk. A family history of the disease, early ankle and hip joint involvement and a higher number of affected joints at disease onset are indicators of poor prognosis in ERA patients (13). Systemic JIA has various prognoses with the majority of patients having a monocyclic disease course. However, articular involvement in sJIA patients usually persists. The remission rate markedly increases and the rate of joint damage decreases along with biological treatment (19). The response to biological treatment is lower in patients with psoriatic arthritis compared to other JIA subtypes (61). The percentage of achieved remission status among patients with RF-positive and RF-negative polyarticular JIA and extended oligoarticular JIA was found to be similar in patients under biological treatment, particularly etanercept. Biological agents have been shown to be efficient and safe in JIA patients, despite reports of increased frequency of infections that sometimes require hospitalization and sporadic reports of autoimmune diseases. There is some evidence that treatment with TNF blockers could increase the risk of malignancy in children. However, a clear causal relationship has not been established since underlying illnesses and the use of concomitant immunosuppressants bear a risk of malignancy as well (62). Frequent depression, sleep disturbance, anxiety and fatigue are reported more commonly among JIA patients than in healthy controls (42). Therefore, psychosocial monitoring could improve the success of treatment. JIA patients are more prone to cardiovascular disease than healthy controls (63). Routine echocardiographic screening improves the disease prognosis.

## CONCLUSION

JIA is a heterogeneous disease group with many controversies regarding treatment options and clinical course. Since diagnosis and treatment delay can lead to irreversible damage, early recognition and adequate, timely treatment are of crucial importance. Disease activity measurements are important tools in terms of treatment success and follow-up. Occasionally, the treatment should be personalized with the protection of general treatment principles. A multidisciplinary approach is important in order to avoid the adverse effects of the drugs. The main goal of the treatment is to prevent possible disease-related complications and to enable patients to live a healthy life.

## Figures and Tables

**Table 1:**
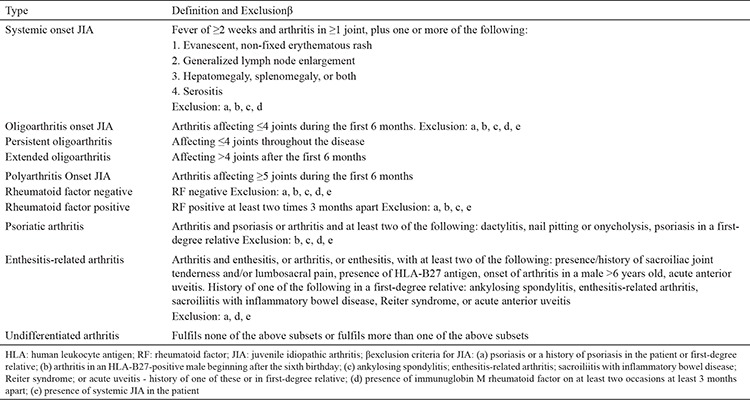
International League of Associations for Rheumatology classification of subtypes of juvenile idiopathic arthritis (4)

**Table 2:**
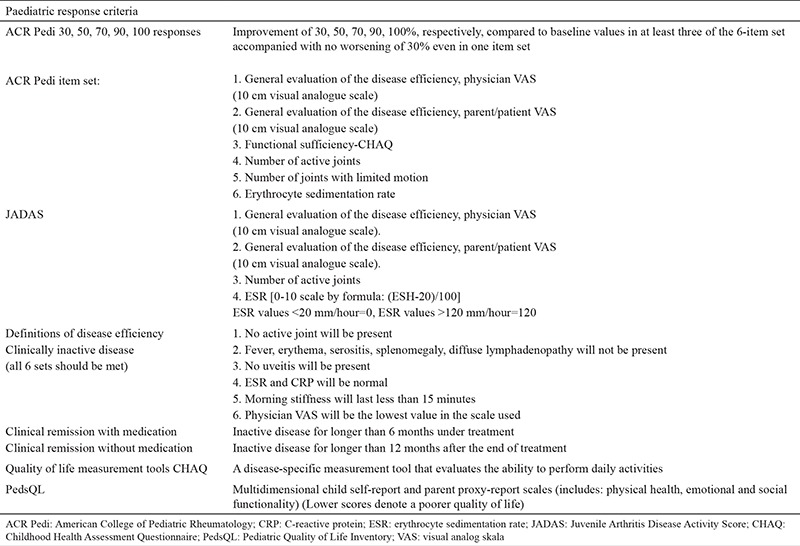
Instruments for measurement of juvenile idiopathic arthritis disease activity routinely used in clinics for paediatric rheumatology (38-44)

**Table 3:**
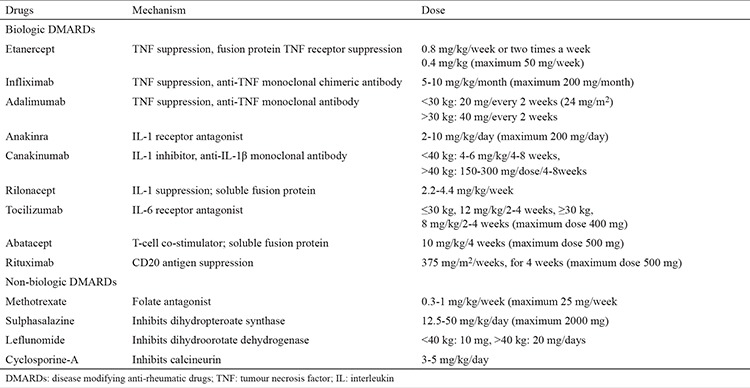
Drugs used in treatment of juvenile idiopathic arthritis (1,5,45)
